# Cholera in Portugal and Madeira

**Published:** 1856-10

**Authors:** 


*Cholera in Portugal and Madeira.*—Lisbon letters speak of Cholera as still continuing its ravages in Lisbon and through the provinces. Peniche is now declared infected. The French screw liner, Prince Jerome, has lost several men from this disease and typhus fever. The intelligence from Madeira is very disastrous. Cholera broke out at sea among some Portuguese troops that left Lisbon on the 20th of June, and when they landed at Funchal a fair was being held, the troops mixed with the inhabitants, and cholera broke out among the latter. When the Avon left Madeira on the 3d of August, there had been 5,000 cases of cholera and 1,500 deaths among the population of Funchal, which numbered only 28,000. The panic at Funchal was terrible; all business was suspended, the shops were closed, and every family isolated itself. The dead lay unburied in the cemetery, and fires were kindled there to mitigate the evil effects arising from the putrefaction of dead bodies. The government at length got twelve men to dig graves, and six of them literally dug their own, for they died almost immediately, and were buried in the graves they had made for others. Only one Englishman, an hotel keeper, had died of it. Seventy English escaped from Madeira in the Avon, and these were all the packet could accommodate. There were about two or three hundred left; but, fortunately, this is not the season for English people to be at Madeira.—*Med. Times and Gaz.*, Aug. 16, 1856.

